# Distanciation as a technology of control in the UK hostile environment

**DOI:** 10.1177/02610183231223952

**Published:** 2024-03-18

**Authors:** JESSICA L. POTTER, ISABEL MEIER

**Affiliations:** 4919 University College London, UK; 5995 Northumbria University, UK

**Keywords:** governmentality, healthcare, hostile environment, migrants, politics of distance

## Abstract

This article considers how distanciation, understood as the active production of different forms of distance as a method of control, is used to manage people racialised and criminalised as migrants within the UK's hostile environment. Analysing different policies introduced under the hostile environment agenda, as well as the more recent New Plan for Immigration, we argue distanciation is a key tactic that shapes these policies and their implementation as well as offers us insight into changing forms of governing migration. Drawing on the analysis of a wide range of policy documents, the paper attends to different forms of distanciation used as a method of control within the UK's wider hostile environment and then presents the results of a case-study of how distanciation is mobilised within the English National Health Service, under the Migrant and Visitor Cost Recovery Programme in particular, which was introduced in 2014 to ensure the NHS receives ‘a fair contribution’ from people racialised as migrants. Addressing different forms of distanciation such as – spatial, legal and emotional – we argue that the lens of distance can offer insights into how detachment – increasing distance between different agents in immigration law and border enforcement is an intentional design to control empathy, solidarity and resistance. Tracing ways these forms of distanciation are designed into legislative and administrative measures helps us better understand how hostile environment policies work as well as locating agencies and possibilities of resistance within different spaces, agents and subjects of bordering.

## Introduction: 10 years of the hostile environment

It has been over a decade since Theresa May, then Home Secretary, announced the objective to create ‘a really hostile environment for illegal migration’ (Kirkup and Winnett, 2012; also see editorial introduction). While the Immigration Acts of 2014 and 2016 marked a significant change in how people racialised as migrants are governed through the state, including a dramatic increase in spaces and agents of everyday bordering (Griffiths and Yeo, 2021; Yuval-Davis et al., 2019), controlling, managing and even punishing migrants through administrative and legislative powers is by no means new. Since the first Alien Act in 1905, immigration policies have functioned as a means to control entry of those racialised as outsiders – first targeting Jewish migrants from Eastern European countries and then, with the Act in 1971, restricting entry, stay and citizenship rights to those from former colonies (Virdee, 2014). We follow many other scholars in viewing the control and management of people racialised and criminalised as migrants through these policies as ongoing, colonial systems of racial ordering (El-Enany, 2020; Mayblin, 2018; Mayblin and Turner, 2020), entangled with different political and economic demands (Gutiérrez Rodríguez, 2021).

In this article we take a closer look at different policies introduced under the hostile environment agenda, including the more recent New Plan for Immigration, and show how discourses and measures around migration and asylum over the last ten years have drawn on a politics of distance to rationalise and govern. Rather than analysing the underlying objectives of the hostile environment which has been done elsewhere (see for example Griffiths and Trebilock, 2022; Griffiths and Yeo, 2021; Webber, 2019), we examine techniques of governmentality; the means by which hostile environment policies are devised and implemented. Here we specifically examine how politics of distance are mobilised as a technology of control. The role of distance in sociology has a long history, arguably beginning with Georg Simmel's analysis of secrecy and the role of strangers which revealed how the negotiation of distance, both metaphoric and geometric, shapes social relationships (Simmel, 1906). In this paper, we speak of distance not solely in geographical terms but as ‘socially, bodily and politically produced’ (Handel, 2018).

The article first situates distance as a technology of control within wider scholarship on governability, bordering and othering. We then briefly discuss our methodological approach before presenting a broad analysis of different modes of distanciation used within the hostile environment policy agenda. To further ground our arguments, we use Potter's decade-long experience as a doctor, researcher and activist in migrants’ experiences of healthcare access, to take a deep dive into the British National Health Service (NHS). In particular, we focus on The Migrant and Visitor Cost Recovery Programme, introduced in 2014, and designed to ensure the NHS receives ‘a fair contribution’ from those racialised as migrants (Department of Health, 2013).

## The politics of distance

This article considers distance as an important lens through which to understand how people racialised and criminalised as migrants are governed under the UK's hostile environment. We add to the literature on the politics of exclusion (Tyler, 2006), criminalisation (Bhatia, 2015; Bosworth and Turnbull, 2014), belonging (Yuval-Davis, 2006), exhaustion (Emejulu and Bassel, 2020), discomfort (Meier, 2020) and unease (Darling, 2011); as well as evidence considering regulation of asylum accommodation and the effects of its privatisation (Darling, 2011, 2016). Our contribution examines how distance is mobilised as a technology of exclusion and control through legal, spatial and emotional means. In this section we want to draw attention to a politics of distance running through a wide range of policies and measures and across different agents, subjects and spaces of bordering, that control possibilities of solidarity, empathy and resistance.

Scholarly debates on the hostile environment over the last ten years have focused on how different mechanisms of ‘everyday bordering’ (Yuval-Davis et al., 2019) produce the ‘hostile environment’. This is outlined in a series of administrative and legislative documents through what Darling (2022), in his work on the politics of discretion, has termed ‘localised measures of hostility, discomfort, and abandonment’. We argue many of these ‘localised measures’ are designed to create spatial, legal and emotional distances. The ways in which these different forms of distance are mobilised through government policies remains largely absent within academic discourse.

Ariel Handel, in the paper ‘Distance matters: mobilities and the politics of distance’, argues scholars across different disciplines have done ‘important work in challenging, nuancing, and complicating the rigid notion of distance – notably, though, without directly using that word’ (Handel, 2018: 475). In his attempt to re-politicise distance, Handel explores the ways in which distance is socially, bodily, and politically produced and highlights how ‘distances are not always measurable or contiguous, but rather based on experience, affect, and language’ (Handel, 2018: 474). Handel's main focus is how mobilities of specific peoples are controlled and managed through the active production of differentiated distances and spatialities – a process he terms ‘distanciation’. In this paper, we consider other domains of distanciation, specifically the mobilisation of emotional and legal distances. We understand spatial distanciation as the increase of physical, measurable distance between different agents, subjects and spaces of the hostile environment. Legal distanciation, we propose, involves the ways in which distances are increased between legal entities of the immigration system. Here we are also interested in the ways the increasing privatisation (Darling, 2016, 2022) and outsourcing to third countries (McKinney et al., 2022) increases legal distances between the state and the applicant through the introduction of mediating third parties. With emotional distanciation we refer to a sense or feeling of not-belonging that is intentionally created.

Our approach to distance is also influenced by Orlando Woods and Lily Kong's work on the latent politics of distance, exploring alternative geographies of migrant religion and how they can lead to the production of latent distances within and between migrant communities (Woods and Kong, 2020). Woods and Kong describe these politics of distance as ‘latent’ as they are ‘subtle and nuanced, and often obfuscated by the overarching logic for separation’ (Woods and Kong, 2020: 349). Here we want to return to Griffith and Yeo's analysis of the hostile environment and their argument that the diffuse and hard to pin down policy basis for its introduction are intentional and play a significant part in how cumulatively a specific and holistic policy strategy was formed (Griffiths and Yeo, 2021). Attending to latent technologies of this policy strategy as well as its follow-up – the New Plan for Immigration – can therefore be used to shed light on its many latent mechanisms of everyday bordering and how it impacts people racialised and criminalised as migrants in a UK context.

## Methodology

Our thinking around the politics of distance first developed through discussions relating to research projects exploring the experiences of migrants living in the UK, one focused on the politics of care (Potter, 2018a) and the other the politics of asylum (Meier, 2018). In this paper, we reveal the ways in which distance is mobilised as a form of control through analysis of a wide range of policy documents, using governmentality as an analytic lens and drawing on our own experiences in activism, migration research and healthcare provision to situate our findings. Our approach is rooted in an overtly anti-racist and de-colonial practice (e.g., de Leeuw et al., 2012), emphasising the potential for resistance and social change through and with research.

Many scholars have emphasised state interests in terms of reinforcing racialised hierarchies and inequalities, as well as the power of governing documents as tools of control (Ahmed, 2004; Cook, 2022; Meers, 2018). We apply Foucault's ‘governmentality’ as a tool which allows us to attend to practices of government and the various ways in which these practices are implicated in how ‘truth’ is produced across different social/political spheres (Burchell et al., 1991). Foucault described government as ‘the way in which the conduct of individuals or of groups might be directed. To govern, in this sense, is to control the possible field of action of others.’ (Foucault, 1982: 789–790). Using governmentality facilitates questions such as ‘Who governs what? According to what logics? With what techniques? Toward what ends?’ (Rose et al., 2006). Rather than focus on the rationalities that underpin the hostile environment this article focuses on *techniques of government* – the means by which particular policies are devised and implemented (Olssen, 2006). In addition, instead of a focus upon top-down discursive practices (such as those inscribed in policy), we add to our analysis the lived experiences of people caught up in the hostile environment, enabling what McKee refers to as ‘realist governmentality’ (McKee, 2009).

First we reviewed relevant policy documents including the Immigration Acts 2014 and 2016, the New Plan for Immigration (HM Government, 2022), the Migration and Economic Development Partnership with Rwanda (McKinney et al., 2022), the Consultation to Improve Arrangements for Asylum Accommodation Dispersal (Home Office, 2022) and the Visitor & Migrant Cost Recovery Programme (Department of Health, 2014). We used thematic analysis (Braun and Clarke, 2006), organising our data specifically to explore how distances are mobilised as a method of control. Our initial analysis revealed several modalities through which distanciation is mobilised: spatial, legislative, and emotional. We then focused on the Visitor & Migrant Cost Recovery Programme in more detail, producing more specific findings regarding these different modalities through which distanciation is used as tactic of governance. This case study included a collection of documents (policies, consultations, impact assessments, guidance and resources) from the Visitor & Migrant Cost Recovery Programme on the UK government website. We specifically focused on the white paper outlining the objectives of the programme – ‘Making a fair contribution’ and the programme's implementation guide (Department of Health, 2015b, 2017). We include parliamentary transcripts and media publications to illustrate our arguments. Alongside these data we bring in our own experiences as activists and researchers in the field of migrants’ rights and Potter's work as a doctor in the NHS.

## Distanciation and the hostile environment

Reviewing hostile environment policies and those that fall under the New Plan for Immigration, reveals not only the continued active production of spatial, legal and emotional distanciation but also an overall increase of distanciation over time. Later in this paper we explore forms of distanciation in relation to the health care service, but first we want to share a few observations about trends in policy design more widely.

### Spatial distanciation: Out of sight out of mind

Spatial distanciation has been used as a tactic of colonial power for centuries (Bernault, 2019; Kalpagam, 2014; Legg, 2006). Hostile environment policies have made increasing and more extreme use of forms of physical distanciation: from the UK's massive so-called immigration ‘detention estate’ (e.g., Silverman et al., 2023), to the plan to set up new efficient ‘one-stop shop’ processing centres, to full dispersal mode of asylum accommodation, to the complete ‘outsourcing’ of asylum application under the new Migration and Economic Development Partnership with Rwanda. These ways of creating spatial distanciation between different racialised migrants and support networks, legal advice centres and community organisations is a tactic to prevent the building of solidarity and resistance movements (City of Sanctuary Local Authority Network, 2022). Research on empathy in particular shows the significant ways spatial distance shapes our relationship, feelings of closeness and ability to understand and share the feelings of others (Schiano Lomoriello et al., 2018).

A report from a meeting of the Committee on Home Affairs in early 2004 reveals that tactics of physical distanciation used to deter, punish and disconnect are by no means new. In an attempt to remove the incentive for ‘economic migrants’ to apply for asylum, the committee considered different ‘segregation schemes’ and policy proposals to ‘ensure asylum seekers are segregated from wider UK society until their applications have been finally determined’ (Committee of Home Affairs, 2004). Furthermore, they proposed ‘more radical options’, specifically ‘A policy of detaining *all* new asylum seekers until their claims are determined’ and ‘an off-shoring processing centre’, which became reality in 2022 (McKinney et al., 2022).

In the first three months of 2022, 25,282 people entered detention in the UK (Home Office, 2022). The ‘deport first, appeal later’ measure in the 2014 and 2016 Immigration Acts stripped thousands of people of the right to an in-country appeal, detaining them for many years (Webber, 2019). Ongoing campaigning against detention centres by activist groups makes visible the awful prison-like conditions, cultures of sexual abuse, racism and general mistreatment within these centres. After the plans to open a first efficient ‘one-stop shop’ processing centre were put on hold by activist campaign work in Spring 2022, within a few months the government announced the Migration and Economic Development Partnership with Rwanda. The partnership involves the UK paying Rwanda an initial £120 million to host those who have ‘illegally’ entered the UK according to the 2022 Borders and Nationality Bill and whose claims for asylum are therefore ‘inadmissible’ under this new piece of legislation. The complete ‘outsourcing’ of asylum applications to Rwanda marks the most extreme form of spatial distanciation and a clear escalation of this tactic over the last 20 years.

### Legal distanciation: Third party involvement and the designed removal of power from local authorities and other state actors

Specific aspects of the immigration system including the administration and running of detention centres have been outsourced to large security companies for several decades (Griffiths and Yeo, 2021). When Harmondsworth, the UK's first detention centre, was opened in 1970 the UK government contracted Securicor, a private security company, which later became part of G4S. Since 1970, third parties have increasingly become involved in the administration, running and enforcement of the immigration system. Over time, the Home Office and other state actors such as local authorities, handed more and more control to the private sector (Darling, 2016; Fee, 2022). Airlines and ferry companies started to become responsible for immigration checks on their passengers through the Immigration Carriers’ Liability Act of 1987 (Harding, 2012). The Asylum and Immigration Act of 1996 made it a criminal offence to employ someone without the right to remain in the UK. The Immigration Acts 2014 and 2016 then marked the largest third-party involvement in immigration control in UK government history. In this section we want to briefly reflect on these trends as tactics of increasing legal distanciation, in which states intentionally hand over legal powers to third parties and private companies to distance themselves from accountability under national law, the Refugee Convention and Human Rights law. The increasing involvement of third parties such as landlords, health care professionals, private companies and schools represent both the internalisation of national borders (Griffiths and Yeo, 2021) and the diffusion of people forced to function as border guards (Yuval-Davis et al., 2019) into different private and social spheres. Here we want to pay attention to the increase in legal distanciation this privatisation produces – between people racialised and criminalised as migrants – and the state.

All asylum accommodation in the UK is provided by private companies, often without any experience in the area. Changes in legislation over the years removed power from local authorities to have oversight, inspection rights or sanction power if housing standards are not met (Fee, 2022). In 2019, 10-year accommodation provision contracts worth £4 billion, were awarded to Serco, Mears Group and Clearsprings Ready Home (Fee, 2022). In April 2022, The Minister for Safe and Legal Migration announced with immediate effect a move to a full model for dispersal, arguing the asylum accommodation is ‘under enormous pressure because of the significant and sustained increase in asylum intake over the last 12 months and the build-up of the population as a result of Covid-19 related measures’. All local authority areas of the UK – England, Scotland and Wales – must participate in the new full dispersal system, under which all authorities are legally required to vacate and house asylum seekers. Until now the private company administrating the housing had to consult the local authority and the local authority had 72 h to consider the request on the ground of safeguarding (Fee, 2022), although the private company could ‘seek permission from the Home Office to override the local authority's objections’ (House of Commons, 2017). As all local authorities are now legally required to accommodate a specific percentage of people, the City of Sanctuary Local Authority Network (2022) have already raised concerns about not having legal powers to scrutinise private provision of accommodation or offer the necessary resources to guaranty safeguarding and support, particularly to vulnerable people.

### Emotional distanciation: Designed alienation

Alienation is often used to describe a feeling or sense of being ‘excluded’, ‘othered’ or ‘out of place’ (TenHouten, 2019) – the opposite or absence of feeling a sense of belonging. Here we want to draw on Yuval-Davi’'s (2006) work on politics of belonging to speak to the different emotional distances actively created. Yuval-Davis differentiates between belonging and the politics of belonging. While belonging is about emotional attachment, about feeling ‘at home’, politics of belonging is ‘the dirty work of boundary maintenance’ (Crowley, 2017). As such, the politics of belonging can be described as a political project of maintaining systems of colonial ordering. Here we want to focus on what boundary-making work hostile environment policies are involved in, specifically highlighting the emotional dimension of the distances created. Drawing on other scholars’ thinking around emotional distances as alienation (Fanon, 2018; Hook, 2004; TenHouten, 2019), we want to look at how hostile environment policies work by generating forms of emotional distanciation across different spaces and agents.

Using Hochschild (1979) and Sirriyeh (2015), Griffiths and Yeo (2021) emphasised how ‘the lack of clear objectives or official monitoring might suggest the policy approach is propelled less by practical considerations around cost, resources and numbers, than “feeling rules” appealing to notions of belonging, fairness and national sovereignty’. The significant escalation of the message of non-belonging becomes apparent when reviewing 10 years of the hostile environment: From 22 of July to 22 of August 2013 a pilot scheme took place, about 1 year after Theresa May's announcement of wanting to create a hostile environment, in which vans drove around six London boroughs carrying the message ‘In the UK illegally? Go home or face arrest’. The scheme was part of Operation Vaken (Jones et al., 2017; Tyler, 2018) and was supposed to encourage people living illegally in the UK to voluntarily return ‘home’. With the Immigration Acts 2014 and 2016 a wide range of services became harder to access for people racialised as migrants and the implicit message ‘you do not belong here’ echoes across people's lives. As highlighted in the editorial introduction, the Windrush scandal also showed racialised citizens can be turned into ‘migrants’ any time (Bhambra, 2018) leaving people with a feeling or sense of never being safe and fully at home. The 2022 Nationality and Borders Act provided the legal basis to strip individuals of their citizenship if they ‘pose as threat to the UK or whose conduct involves very high harm’. The Institute of Race Relations (IRR) recently published a report showing these powers are almost exclusively used on Muslims, mostly of south Asian heritage (Webber, 2022). This escalation of emotional distanciation through the use of administrative and legislative measures by the UK government not only targets people not born in the UK but anyone racialised as permanent outsiders (Virdee, 2014).

This section explored different forms of distanciation used as a method of control within the British hostile environment more widely, next we present a case study exploring how spatial, legal and emotional distanciation are mobilised within the English NHS guided by the Migrant and Visitor Cost Recovery Programme.

## Distanciation within and through the NHS – an exclusionary politics of care

The UK NHS, founded in 1948, was designed to deliver healthcare based on clinical need and not ability to pay. The NHS is funded through general taxation and national insurance contributions with access free at the point of service. In the early years of the British welfare state, concerns people might travel to the UK in order to ‘access everything they need’ were raised, eventually resulting in the introduction of legislation to restrict access to the NHS for people not usually resident in the UK (Potter, 2018b). While the NHS was founded upon the idea it would be freely accessible to all, it has been restricted to those who are ‘ordinarily resident’ in the UK. This slippery legal concept has changed over the years but at the time of the first NHS Charging regulations in 1982, anyone who lived in the UK as the regular order of their life could consider it their usual residence. Over time, across a number of Immigration Acts, and their accompanying amendments to the NHS Charging Regulations, increasing groups of people have been excluded including: students; people moving to work in the UK but without indefinite leave to remain; and failed asylum seekers.

A key focus of the Conservative party, elected as part of a coalition government in 2010, was reducing net migration. Immigration featured increasingly in the media at this time, including representations of people racialised as migrants using the NHS as ‘health tourists’ (Jayaweera, 2010). Despite legislation existing to facilitate charging of overseas visitors and those racialised and criminalised as migrants, as a parliamentary transcript reveals, ‘there were then three decades when very little happened’ (House of Commons Committee of Public Accounts, 2017).

In 2013, a government consultation was launched into migrant and visitor charging (Department of Health, 2013) justified by it being ‘widely recognised that the NHS has a longstanding weakness in charging foreign nationals who use the NHS’ and that the current system was ‘very generous’ as it allowed people living temporarily in the UK free NHS care ‘without contributing’ as well as providing free access to General Practice for all, including tourists (Department of Health, 2013). Whilst the consultation acknowledged most people racialised as migrants and visitors make ‘only occasional and necessary use of the NHS’, it also suggested ‘health tourists’ – ‘people who take advantage of our current generous entitlements’ – were attracted by the open system (Department of Health, 2013).

The objectives of the Visitor and Migrant Cost Recovery programme were set out in a forward to the paper ‘Making a fair contribution’ by then Health Secretary Jeremy Hunt (Department of Health, 2015b). The aim was to ‘make sure only those who live here and contribute financially receive free NHS care’ to be ‘fair to the British taxpayer’ and ‘improve sustainability’. This statement immediately positions those who do not pay tax (such as people unable to work due to disability or caring roles) as undeserving of healthcare and a drain on NHS resources. In addition, there is an inherent assumption migrants do not contribute financially at all whether through consumptive taxes or income tax.

The NHS Visitor and Migrant Cost Recovery Programme Implementation Plan 2014–2016 set out the policy and legislative agenda in more detail (Department of Health, 2014). Phase 1 was designed to improve existing systems: instituting incentives for NHS Trusts to recover costs from patients by increasing the chargeable tariff from 100 percent of cost to 150 percent; and sanctions for Trusts who failed to identify and charge people ineligible for free NHS care. Phase 2 introduced additional mechanisms to identify chargeable patients including: a data sharing agreement between the Home Office and the NHS; the introduction of an electronic banner, highlighting chargeable status on the NHS IT system; and changes to primary and secondary care registration forms to capture information relating to immigration status. Phase 3 brought in the Immigration Health Surcharge (HIS). This flat rate charge for healthcare is levied in association with the visa application process for people seeking to move to the UK from outside the EEA for longer than 6 months. This charge is between £470 and £624 per year of the duration of the individual's visa. Phase 4 detailed a plan to extend charging beyond secondary care and into primary care and A&E which has not yet occurred.

Examining these policies in detail, we illustrate ways in which different forms of distanciation are used to control the field of possible actions of people both subject to bordering and embroiled in upholding bordering practices in the NHS. Across the pandemic, as the practice of social-distancing was embedded into the everyday, reliance on distant modes of governing became more visible. We show how creating physical space between people employed to implement the NHS charging regulations – overseas visitor managers (OVMs) – and people they identify as chargeable has led to a reliance on digitally encoded identifiers of difference which serve both to improve efficiency and amplify discrimination. Moving on to legal distanciation, as we have shown in broader policy arenas, we reveal how this separates the state from its duty of care to migrants and others racialised as foreign. Finally, we focus on emotional distanciation and the use of alienation not only to produce people undeserving of healthcare but to limit spaces for resistance.

## Spatial – COVID and distant modes of governing

Most charging for healthcare in the NHS occurs in secondary care – hospitals, maternity units and outpatient departments. Identification of potentially chargeable individuals occurs through a number of mechanisms: with a ‘baseline’ screening question ‘Where have you lived in the last 6 months?’ (Department of Health, 2017). This question might be posed via a form handed out at a clinic appointment or directly by OVMs, administrators or other patient-facing staff. OVMs might be alerted to potentially chargeable patients through referral to the overseas team in the hospital by healthcare workers or administrative staff who suspect a patient might be chargeable; or through digital identification systems such as the banner system on the NHS spine or overt searching of in-patient databases (Department of Health, 2017).

Section 11.21 of the implementation guide states:

*‘In some departments that cater for the very elderly or those with mental health problems, or when direct admission from critical healthcare is needed, the baseline questioning may be inappropriate or unworkable. In these cases, admissions staff should still be aware of the possibility of people being liable for charges, and should notify the OVM of any patient whose chargeable status is unknown based on any non-discriminatory information they have (i.e., not purely on the basis of appearance, language, accent etc.).’* (Department of Health, 2017)

Use of the phrase ‘not purely’ implies it is acceptable to use this information so long as it is not used in isolation. Given people from white ethnic backgrounds who grew up in the UK but no longer live there may not be entitled to free NHS care, even the mention of the use of appearance, language or accent here is discriminatory. Prior to the pandemic, an OVM interviewed anonymously for a BBC programme on the topic stated one method of targeting potentially chargeable patients included searching hospital data for ‘potentially foreign sounding names’ (Nye, 2019).

During the pandemic, many staff who were not directly involved in patient care moved away from the frontline in order to reduce risk of contracting COVID-19. This included OVMs who are responsible for implementing the NHS charging regulations. Creating distance between patients and OVMs on the face of it might reduce the number of people charged. However, moving away from the frontline forces an increased reliance on distant modes of governing – including the use of digital identifiers of difference such as new NHS numbers or foreign sounding names.

In 2019, according to a freedom of information request submitted to several NHS Trusts, a new digital system called the Message Exchange for Social care and Health (MESH) was being rolled out to support the ‘quick & easy, and en mass way to identify potentially chargeable patients’ (NHS England, 2021). Rather than running all hospital admissions or new referrals through the system, there is a suggestion of using a ‘funnel’ to ‘reduce the records to a manageable number’ (Yeoville District Hospital NHS Foundation Trust, 2020). Each overseas team can select their own criteria but examples provided include filtering by records with no GP, no NHS number and no documented address. However, there are clear ethnic disparities among people experiencing homelessness with minoritised ethnic communities disproportionately affected (Wilson and Barton, 2021).

Benjamin (2019) has shown how supposedly sanitised data and ‘objective’ questions reproduce and proliferate racism. Rapid searching of digital data is far more efficient than relying on self-reported information regarding chargeability or networks of NHS workers reporting patients and relies less on the change in culture required to improve charging which we elaborate upon later. Similarly, the issuing of bills after a patient has left the hospital renders the act invisible to clinicians who might protest. While this shift to more distant, digitised modes of governing was present in hospitals before, the COVID-19 pandemic accelerated their use and their efficiency.

## Legal distanciation: The primacy of charging

When asked for comment after issuing a series of large bills to a patient unwell in intensive care, one NHS Trust responded by stating its legal obligations to follow the guidelines (Nye, 2019). This argument allows healthcare providers to distance themselves from protests that they should be delivering care in line with other moral and legal obligations such as the Equality Act 2010 or the Human Rights Act 1998.

The obligation to identify and charge people ineligible for free NHS care was extended in 2015 to providers of all ‘relevant services’ signalling the expansion of the healthcare charging regime from ‘NHS services’ to private and voluntary providers delivering other provisions (including, for example accommodation) under the remit of the National Health Service Act 2006. A further amendment to the charging regulations in 2017 added greater distance between the state's healthcare provision for some migrants and their human right to health by stipulating that care could be withheld in lieu of payment for all non-urgent conditions (Bulman, 2017). For the first time in the history of the NHS, care could legally be denied to people who could not afford to pay upfront. This resulted not only in those *in*eligible for free NHS care being denied potentially life-prolonging treatment such as chemotherapy, but extended to others, racialised as foreign – most notably people from the Windrush Generation (Gentleman, 2018).

Additional important differences within the legal entitlements to healthcare for people racialised as migrants and visitors broadly define who is un/deserving of care. For example, there is an extensive list of diseases exempt from charging – almost all are contagious (malaria the exception) underscoring the importance of public health. This is problematic for two reasons. First, people do not walk around with their diagnosis on a sign around their necks – healthcare access and the process of diagnosis is a complex, social process built upon trust between healthcare provider and care-seeker, undermined by hostile environment policies and practices (Chase et al., 2017; Potter et al., 2020). Second, the pandemic increased visibility of ‘public health’ as a target of government. COVID-19 made stark the fact that our health as individuals is reliant on our health as communities. There was a sense that anyone could get COVID and at first, we were all in it together. Contrary to neoliberal and hostile environment logics, under which some people are more deserving of healthcare than others, when anyone can be a risk to themselves or anyone else, everyone must be offered access to healthcare.

This justification – that healthcare is an important aspect of public health – has been used by campaigners to call for universal access to healthcare and #VaccinesForAll. However this argument positions the worth of individuals (who would otherwise be excluded from healthcare services) as only valuable because without care they become a potential threat to others normally included within the health system. This form of necropolitics (Mbembe, 2003) also speaks to Agamben's work: The pandemic is another state of exception within which people racialised as migrants came to be included within legislation entitling them to care solely because to exclude them would threaten political life (Agamben, 1998).

## Emotional distanciation – producing alienation

Healthcare access is a social process of which a key component is recursivity –health seeking behaviour and health service interactions are impacted by previous experiences (Dixon-Woods et al., 2006; McEvoy et al., 2017). Discriminatory identification processes or receiving a bill after returning home from the hospital are therefore likely to act as a deterrent to future attempts to access healthcare. In addition to hostile practices, differences in eligibility characteristics further differentiate deservingness relating to free NHS care. One place where this is particularly visible is the Immigration Health Surcharge which, once paid allows an individual to access the NHS on the same basis as a British citizen usually resident in the UK. The single exception to this entitlement is assisted conception which is not provided for surcharge payers – governing who can and cannot procreate, creating hierarchies of life (Department of Health, 2017).

Emotional distanciation is not only used to control people racialised as migrants but those embroiled in upholding the hostile environment. Improving identification of chargeable individuals is one of the key aims of the programme. One important strategy required to do this was a need to ‘change culture’:

*‘What we want – it is not just about this scheme, although it goes for this scheme as well - is a culture in the NHS where everybody who works in it feels not only the responsibility we want them to have for patient care and so on but also a financial rigour responsibility. That is the thing we want most of all from trusts’* (House of Commons Committee of Public Accounts, 2017).

This is also reflected in the implementation guide where an aim ‘to encourage a culture change so that a patient's eligibility for free NHS care is checked more regularly than is currently the case’ is accompanied by a plan to evaluate culture change as a measure of progress (Department of Health, 2015a). The need to do *work* to encourage identification and charging within the NHS highlights how far removed this is from existing culture exemplified by the founding principle of the NHS – a health service designed to deliver care free at the point of service, based on clinical need and not ability to pay (Bevan, 1952). In order to overcome the possibility that through upholding the charging regulations ‘the moral oddity of one's action will […] be discovered, and once discovered, made into a painful moral dilemma’ (Bauman, 1991: 159) it is necessary to create emotional distance between healthcare workers and the institutional values (of universalism and non-discrimination) they embody.

NHS workers have experienced years of stagnant wages which have fallen behind inflation resulting in some nurses having to rely on food banks (Dean and Longhurst, 2017; Raven, 2023). There is a national recruitment and retention crisis with 1 in 10 posts unfilled (Campbell, 2022). More is being demanded for less and as working conditions deteriorate, workers lack the resource to provide care to an acceptable standard resulting in moral distress (Jameton, 1993; Reynolds and Mitchell, 2019). When the pandemic hit, health workers’ status was elevated to that of heroes. This positioning produces them as morally responsible for ‘stepping up’ or ‘going above and beyond’ with an accompanying assumption they were not already doing so. This has led to feelings of resentment. These experiences create emotional distance between workers and NHS values alongside compassion fatigue directed towards patients. Framed alongside a migrant crisis with a focus on health tourists who abuse NHS services, working in an under resourced NHS reinforces a narrative that ring fencing NHS services is a reasonable course of action.

For migrant workers, on whom the NHS depends, but who are also subject to hostile environment policies, insecure immigration status and exorbitant visa and health surcharge fees evoke additional anger. One woman, for example, reported to journalists feeling ‘emotionally drained’ by her experiences working as a nurse during the pandemic and that these emotions were compounded by additional ‘anxiety’ over her right to remain (Ray, 2021). The hero status of health workers during the pandemic created space to call for indefinite leave to remain and exemption from surcharge fees for healthcare workers, particularly doctors and nurses. Priti Patel, then Home Secretary said ‘Our offer of free visa extensions shows how our country values the contributions of these heroes.’ (Home Office and Department of Health, 2021) However, the visa extensions only held precarity at bay for a year and the offer did not extend to all workers in the health service – excluded for example were those whose jobs have been outsourced – for example hospital porters and security teams. This hierarchical valuing of jobs is not new, but it does create emotional distance between groups of people racialised as migrants diminishing possibilities for solidarity.

## Conclusion and discussion

In conclusion, we argue the increasing distances between different agents in immigration law and border enforcement is an intentional design to control empathy, solidarity and resistance between different subjects and objects of bordering. Tracing ways these forms of distanciation are designed into legislative and administrative measures helps us better understand how hostile environment policies work while also locating agencies and possibilities of resistance within different spaces, agents and subjects of bordering. In this paper, we showed the various ways spatial, legal and emotional distances are mobilised through different administrative and legislative documents. Close examination of the Visitor & Migrant Cost Recovery Programme revealed how use of distance as a means of control has proliferated through the pandemic in spite of an increased prominence of arguments that access to healthcare is a public good.

Our findings also demonstrate the importance of attending to the ways in which these different modalities of distanciation are entangled. Existing scholarship has pointed at the ways in which spatial distances influence our experience of emotional distanciation (Schiano Lomoriello et al., 2018). Similarly physical distanciation or distant governance can be used to enforce legal distanciation. Governmentality, as an analytical tool to study the ways in which the hostile environment is enforced through different means of distanciation, has allowed us to focus on the discursive practices of hostile environment policy as well as the experiences of those engaged within and through these hostile policies, echoing the importance of what McKee called ‘realist governmentality’ (McKee, 2009). Realist governmentality focuses on ‘examining particular mentalities of rule in their local context’ (McKee, 2009: 467) and by doing so, offers us tools to make visible the effects of government practices within situated experiences and knowledges as well as to locate agencies and possibilities of resistance.

## Funding

The author(s) disclosed receipt of the following financial support for the research, authorship, and/or publication of this article: Research undertaken by Jessica Potter as part of her PhD was funded by the Medical Research Council as part of a strategic skills doctoral research fellowship (grant number MR/M014517/1). Research undertaken by Isabel Meier is funded by the Leverhulme Trust (grant number ECF-2021-664).
